# Beyond Side-Specific Distributions: Patient-Level Bilateral Agreement of Mandibular Third-Molar Classifications

**DOI:** 10.3390/jcm15197362

**Published:** 2026-09-22

**Authors:** Mehmet Fuat Kılıç, Metehan Ünal, Fatıma Zehra Ünal, Semih Gönç, Mehmet Emre Yurttutan

**Affiliations:** 1 Department of Oral and Maxillofacial Surgery, Faculty of Dentistry, Ankara University, 06560 Ankara, Türkiye; semihgonc7@gmail.com (S.G.); memreyurttutan@yahoo.com (M.E.Y.); 2 School of Computer Science and Mathematics, Keele University, Keele ST5 5BG, UK; m.unal@keele.ac.uk; 3 Optoelectronics and Electro-Optics Technologies Programme, Akyurt Vocational School, Ankara University, 06750 Ankara, Türkiye; fzkilic@ankara.edu.tr

**Keywords:** mandibular third molar, bilateral agreement, panoramic radiography, Winter classification, Pell–Gregory classification, Pederson difficulty index

## Abstract

**Background/Objectives:** The right and left mandibular third molars of the same patient may be expected to have similar radiographic characteristics. This study aimed to determine whether side-specific distributions of mandibular third-molar classifications correspond to patient-level bilateral agreement and whether identical Pederson scores may conceal differences in their underlying radiographic components. **Methods:** This retrospective cross-sectional study included 800 mandibular third molars from 400 patients with bilateral mandibular third molars. Panoramic radiographs were evaluated separately for each side using the Winter classification, Pell–Gregory classification, and Pederson difficulty index. Bilateral agreement was assessed using raw agreement, Cohen’s kappa, and weighted kappa coefficients, while side-specific distributions were compared using marginal homogeneity tests. **Results:** Raw agreement was 59.75% for Winter angulation, 56.0% for the combined Pell–Gregory classification, 69.5% for the Pederson difficulty group, and 48.75% for the total Pederson score. Kappa values were 0.42, 0.44, and 0.43 for Winter angulation, the combined Pell–Gregory classification, and the Pederson difficulty group, respectively. Complete agreement across all three systems was observed in 170 patients (42.5%). The Pell–Gregory ramus relationship showed substantial agreement (κ = 0.64), whereas depth showed moderate agreement (κ = 0.41). Left-side Pederson scores were significantly higher than right-side scores (*p* = 0.013), although the mean difference was only 0.15 points. **Conclusions:** Bilateral mandibular third molars showed only moderate patient-level agreement. Similarity in category distributions at the sample level should not be interpreted as within-patient agreement, and identical Pederson scores may conceal differences in angulation and position. Each mandibular third molar should therefore be evaluated separately, particularly in cases close to classification thresholds.

## 1. Introduction

Mandibular third molars are among the most frequently impacted teeth and are commonly managed in oral and maxillofacial surgery practice [1]. Although they may remain asymptomatic for many years, they can be associated with pericoronitis, caries or root resorption in adjacent teeth, periodontal bone loss, and the development of odontogenic cysts or tumors. Their removal may lead to inferior alveolar or lingual nerve injury, mandibular fracture, excessive bone loss, and other intraoperative or postoperative complications. Contemporary management of impacted mandibular third molars requires individualized assessment of clinical findings and anatomical relationships, with coronectomy representing an alternative to complete extraction in selected cases at high risk of inferior alveolar nerve injury [2]. Therefore, careful preoperative assessment is essential for appropriate treatment planning [3,4].

Accurate assessment of the position of mandibular third molars is essential for predicting surgical difficulty and evaluating the risk of potential complications. Panoramic radiography is the primary imaging method routinely used for this purpose because of its low radiation dose, wide availability, and broad anatomical coverage [1,5]. On panoramic radiographs, the Winter classification is commonly used to describe tooth angulation [6], while the Pell–Gregory classification defines the relationship of the tooth to the anterior border of the ramus and its depth [7]. The Pederson difficulty index, which is calculated by assigning and summing scores for the components of these two systems, is widely used to predict surgical difficulty [1,8,9].

Studies on mandibular third molars have mainly focused on preoperative radiographic assessment, estimation of surgical difficulty, and prediction of complication risk [10,11]. The Winter and Pell–Gregory classifications are the most used systems, while the Pederson difficulty index serves as an additional radiographic tool for estimating surgical difficulty. The angulation of bilateral mandibular third molars has also been associated with mandibular arch parameters, including arch length, intermolar width, anterior crowding, and incisor inclination [12]. Because such arch-level factors may affect both sides, the right and left third molars in the same patient might be expected to have similar radiographic characteristics. Previous bilateral studies have reported side-specific category distributions or simple proportions of matching angulation and depth [3,13,14]. However, similar marginal distributions do not necessarily indicate that the two teeth of the same patient fall into the same category. Patient-level agreement corrected for chance has not been comprehensively characterized across the Winter, Pell–Gregory, and Pederson systems. It also remains unclear whether identical composite Pederson scores may conceal differences in their underlying angulation and positional components.

This study aimed to quantify patient-level agreement between the right and left mandibular third molars using the Winter, Pell–Gregory, and Pederson systems and to determine whether similarity in side-specific classification distributions reflects agreement within individual patients. Agreement was also assessed separately for the ramus relationship and depth components of the Pell–Gregory classification. The distributions of classification categories and Pederson difficulty scores were compared between the two sides, and the categories in which bilateral disagreement most frequently occurred were identified. We also determined the proportion of patients who showed bilateral agreement across all three assessment systems.

## 2. Materials and Methods

### 2.1. Study Design

This retrospective study was conducted using panoramic radiographs obtained from the archives of the Department of Oral and Maxillofacial Surgery, Faculty of Dentistry, Ankara University. Patient records from October 2025 to March 2026 were reviewed retrospectively, and eligible patients were consecutively included. No a priori sample size calculation was performed because all consecutive patients meeting the eligibility criteria during the predefined study period were included. All consecutive patients who met the eligibility criteria during the study period were included. A total of 800 mandibular third molars from 400 patients with bilateral mandibular third molars were evaluated. Patient age and sex were recorded, and radiographic data for the right and left mandibular third molars were collected separately. The study was reported in accordance with the Strengthening the Reporting of Observational Studies in Epidemiology (STROBE) guidelines [15].

Ethical approval was obtained from the Ethics Committee of the Faculty of Dentistry, Ankara University (Meeting No. 20/5; Approval Date: 30 March 2026). The study was conducted in accordance with the principles of the Declaration of Helsinki.

### 2.2. Inclusion and Exclusion Criteria

Patients with bilateral mandibular third molars were included if both teeth could be evaluated on panoramic radiographs using the Pell–Gregory classification, Winter angulation classification, and Pederson difficulty index. Eligibility was based on radiographic assessability rather than clinically determined eruption status. No intraoral examination was performed as part of this retrospective radiographic study, and clinical eruption status was not recorded as a separate variable.

The radiographic presence of all 32 permanent teeth, including all four third molars, was required for inclusion. Patients with any permanent tooth missing from either the maxillary or mandibular arch were excluded. Unerupted or impacted teeth visible on the radiograph were considered present. This study-specific criterion was adopted to facilitate consistent tooth identification and minimize potential ambiguity associated with incomplete dentitions. Other exclusion criteria were suspected pathological findings on panoramic radiographs, insufficient image quality for radiographic assessment, or incomplete demographic or radiographic data.

### 2.3. Radiographic Assessment and Classification

All panoramic radiographs included in the study were obtained according to the clinic’s routine protocol using a Planmeca ProOne digital panoramic imaging system (Planmeca Oy, Helsinki, Finland) and the manufacturer’s standard adult panoramic program (64 kV, 5 mA, 10 s). Before the main assessment, two oral and maxillofacial surgery residents, each with four years of clinical experience, jointly reviewed example panoramic radiographs and discussed the application of the Winter classification, Pell–Gregory classification, and Pederson difficulty index to establish a common understanding of the assessment criteria. This preparatory exercise did not include a formal quantitative assessment of interobserver agreement. During the main assessment, the two examiners reviewed each panoramic radiograph together and recorded a consensus classification for each tooth. Cases in which consensus could not be reached were referred to a faculty member with 15 years of clinical experience in oral and maxillofacial surgery, whose assessment determined the final classification. Separate independent ratings were not recorded; therefore, interobserver reliability could not be calculated.

The right and left mandibular third molars were evaluated separately on each panoramic radiograph. The Pell–Gregory classification, Winter angulation classification, and Pederson difficulty index were recorded for both teeth. The Pell–Gregory classification was used to assess the depth of the mandibular third molar relative to the occlusal plane of the adjacent second molar and its relationship to the anterior border of the mandibular ramus. The Winter classification was used to determine the angulation of the mandibular third molar relative to the adjacent second molar. The Pederson difficulty index was calculated by summing the scores assigned to Winter angulation and the depth and ramus relationship components of the Pell–Gregory classification. The resulting score was used to compare bilateral agreement in composite radiographic characteristics rather than to predict or validate actual surgical difficulty across different eruption states. The radiographic classification criteria and the scoring system used to calculate the Pederson difficulty index are presented in Table 1.

### 2.4. Assessment of Bilateral Agreement

The right and left mandibular third molars were compared using the Pell–Gregory classification, Winter angulation classification, and Pederson difficulty index. Bilateral agreement was defined as both teeth in the same patient having the same category. For the combined Pell–Gregory classification, agreement required exact concordance in both the Rasmus relationship and depth components. For the Winter classification, agreement required the same angulation category and the Pederson index; agreement required the same total score. Agreement for the Pederson index was also evaluated by difficulty category (easy, moderate, or difficult). Patients with agreement across all three systems were considered to have complete bilateral agreement.

### 2.5. Statistical Analysis

No missing data were present in the final dataset; therefore, no missing-data procedure was required.

All analyses were performed at the patient level, with the right and left mandibular third molars of each patient treated as paired observations (*n* = 400). Continuous variables were summarized using the mean ± standard deviation (SD), median, and interquartile range (IQR), while categorical variables were presented as numbers and percentages. The normality of continuous variables and paired differences was assessed using the Shapiro–Wilk test.

The difference between the right and left side Pederson scores was assessed using the Wilcoxon signed-rank test because the paired differences were not normally distributed. Agreement between the two sides was evaluated for each classification system using the raw percentage agreement and Cohen’s kappa (κ), which corrects for agreement expected by chance. Unweighted kappa was calculated for the nominal Pell–Gregory and Winter classifications. For the ordinal Pederson score, linear and quadratic weighted kappa coefficients were calculated in addition to unweighted kappa. Unweighted and linear weighted kappa coefficients were reported for the Pederson difficulty categories. Kappa coefficients were also calculated separately for the ramus relationship and depth components of the Pell–Gregory classification. The 95% confidence intervals (CIs) for all kappa coefficients were obtained using percentile bootstrap analysis with 2000 resamples. The marginal distributions of Winter angulation, Pell–Gregory ramus relationship and depth categories, and Pederson difficulty categories were compared between the right and left sides using the Stuart–Maxwell test of marginal homogeneity [16]. Kappa values were interpreted according to the criteria proposed by Landis and Koch: <0.20, slight; 0.21–0.40, fair; 0.41–0.60, moderate; 0.61–0.80, substantial; and 0.81–1.00, almost perfect agreement [17]. These categories were used as descriptive conventions rather than absolute thresholds of clinical importance. Interpretation also considered the numerical kappa estimates, their 95% confidence intervals, raw agreement, and the observed patterns of disagreement. All tests were two-sided, and *p* < 0.05 was considered statistically significant.

In this study, the Python programming language (Python 3.12.6) and pandas (3.0.3), numpy (2.4.6), matplotlib (3.11.0), SciPy (1.17.1), and scikit-learn (1.9.0) libraries were used for data analysis and visualization.

### 2.6. Use of AI-Assisted Tools

During the preparation of this work, the authors used an AI-assisted tool solely for language editing. The authors subsequently reviewed and edited the manuscript and take full responsibility for its content.

## 3. Results

### 3.1. Study Population and Demographic Characteristics

A total of 580 patient records were screened for eligibility. Of these, 180 were excluded based on the predefined exclusion criteria: 88 due to missing mandibular third molars, 48 due to incomplete demographic data, 32 due to inadequate radiographic quality, and 12 due to the presence of pathology affecting the region of interest. The remaining 400 patients were included in the final analysis.

Of the 400 patients included in the study, 268 (67.0%) were female and 132 (33.0%) were male. Patient age ranged from 11 to 48 years, with a mean age of 22.2 ± 4.4 years and a median age of 22 years (IQR, 19–24). Most patients were aged 30 years or younger: 150 (37.5%) were aged 20 years or younger, 230 (57.5%) were aged 21–30 years, 19 (4.75%) were aged 31–40 years, and only 1 (0.25%) was aged 41 years or older (Table 2).

### 3.2. Distribution of Winter, Pell–Gregory, and Pederson Classifications by Side

The most common Winter angulation on both sides was mesioangular (45.25% on the right and 38.25% on the left). This was followed by vertical (27.0%) and horizontal (22.5%) angulations on the right and horizontal (28.25%) and vertical (26.5%) angulations on the left. Distoangular angulation was the least common on both sides (5.25% on the right and 7.0% on the left). According to the Pell–Gregory classification, most teeth were classified as Class II in terms of ramus relationship (69.75% on the right and 65.25% on the left) and as Position A or B in terms of depth. The most common combination was Class IIB (33.75% on the right and 31.0% on the left), followed by Class IIA (30.25% on the right and 27.75% on the left). The proportion of teeth classified as Class III was 19.25% on the right and 22.25% on the left. According to the Pederson difficulty index, most teeth were classified as moderately difficult (62.25% on the right and 59.5% on the left). The proportion classified as difficult was higher on the left (31. 25%) than on the right (27.0%). The distributions for the right and left sides are presented in Table 3.

Comparison of the marginal category distributions between the right and left sides using the Stuart–Maxwell test showed a significant difference in Winter angulation (χ^2^ = 12.60; df = 3; *p* = 0.006). Mesioangular angulation was more common on the right, whereas horizontal angulation was more common on the left. No significant differences were found between the two sides in the distributions of Pell–Gregory ramus relationship (χ^2^ = 4.77; df = 2; *p* = 0.092), Pell–Gregory depth (χ^2^ = 1.74; df = 2; *p* = 0.419), or Pederson difficulty category (χ^2^ = 4.00; df = 2; *p* = 0.135).

### 3.3. Comparison of Pederson Difficulty Scores Between Sides

The mean Pederson score was 5.94 ± 1.17 for the left mandibular third molars (median, 6; range, 3–10) and 5.79 ± 1.14 for the right mandibular third molars (median, 6; range, 3–9). The paired differences between the two sides were not normally distributed (Shapiro–Wilk W = 0.901; *p* < 0.001). The Wilcoxon signed-rank test showed that Pederson scores were significantly higher on the left than on the right (*p* = 0.013). However, the mean difference was only 0.15 points, and the corresponding rank-biserial correlation indicated a small effect (r_rb_ = 0.19). At the patient level, the Pederson score was higher on the left in 119 patients (29.75%), higher on the right in 86 patients (21.5%), and equal on both sides in 195 patients (48.75%).

### 3.4. Bilateral Agreement Between Left and Right Mandibular Third Molars

The raw agreement rates between the right and left mandibular third molars were 69.5% (278/400) for the Pederson difficulty category, 59.75% (239/400) for Winter angulation, 56.0% (224/400) for the combined Pell–Gregory classification, and 48.75% (195/400) for the total Pederson score. Complete bilateral agreement across Winter angulation, the Pell–Gregory classification, and the Pederson score was observed in 170 patients (42.5%). In other words, the right and left mandibular third molars differed in at least one assessment system in 230 patients (57.5%) (Table 4, Figure 1).

After correction for chance agreement, Cohen’s kappa was 0.44 (95% CI, 0.37–0.50) for the combined Pell–Gregory classification, 0.42 (95% CI, 0.35–0.48) for Winter angulation, and 0.43 (95% CI, 0.35–0.51) for the Pederson difficulty category. According to the Landis and Koch criteria, all three values indicated moderate agreement. For the total Pederson score, unweighted kappa indicated fair agreement at 0.32 (95% CI, 0.26–0.39). When the distance between scores was considered, linear weighted kappa was 0.42 (95% CI, 0.36–0.49) and quadratic weighted kappa was 0.52 (95% CI, 0.44–0.59), both indicating moderate agreement. Consistent with these findings, the right and left Pederson scores were either identical or differed by only 1 point in 83.25% of patients (333/400). A difference of 2 or more points was observed in only 16.8% (Figure 2d). However, among the 195 patients with identical total Pederson scores on both sides, 25 (12.82%) differed between sides in Winter angulation, the combined Pell–Gregory classification, or both.

When the two components of the Pell–Gregory classification were examined separately, bilateral agreement differed considerably between them. For ramus relationship (Classes I–III), raw agreement was 82.5% and kappa was 0.64 (95% CI, 0.56–0.72), indicating substantial agreement. For depth (Positions A–C), raw agreement was 62.75% and kappa was 0.41 (95% CI, 0.34–0.49), indicating moderate agreement. Thus, a large proportion of the right–left disagreements in the combined Pell–Gregory classification involved the depth component. Of the 176 discordant pairs, 73 (41.5%) differed only between Positions A and B within the same ramus class (IIA–IIB) (Figure 2b).

Disagreements in Winter angulation were also concentrated between specific categories. Of the 161 discordant pairs, 76 (47.20%) involved a mesioangular tooth on one side and a vertical tooth on the other, while 34 (21.11%) involved a mesioangular–horizontal combination. Extreme combinations, such as distoangular angulation on one side and horizontal angulation on the other, were uncommon and occurred in only seven patients (Figure 2a). For the Pederson difficulty categories, 115 of the 122 discordant pairs (94.26%) involved adjacent categories (easy–moderate or moderate–difficult). An easy–difficult discrepancy was observed in only seven patients (1.75% of the total sample) (Figure 2c).

Across the assessment systems, raw agreement was highest for the Pederson difficulty category and lowest for the total Pederson score. After correction for chance agreement, kappa values were similar for Winter angulation, the Pell–Gregory classification, and the Pederson difficulty category (0.42–0.44), with all three indicating moderate agreement.

## 4. Discussion

This study evaluated radiographic agreement between the right and left mandibular third molars using Winter angulation, the Pell–Gregory classification, and the Pederson difficulty index. Kappa coefficients for the main assessment systems ranged from 0.42 to 0.44, indicating moderate agreement between the two sides. Complete bilateral agreement across all three assessment systems was observed in only 42.5% of patients. In other words, 57.5% of patients had at least one difference between the right and left mandibular third molars in terms of angulation, anatomical position, or radiographic difficulty. These findings suggest that the radiographic characteristics of a mandibular third molar on one side should not be assumed to apply to the contralateral tooth and that each tooth should be evaluated separately in bilateral cases.

Bilateral symmetry of mandibular third molars has been investigated in only a limited number of studies. Litonjua [13] reported symmetry in both depth and angulation in only 27% of 63 patients with bilateral impacted mandibular third molars. Akarslan and Kocabay [3] compared symptoms, pathological findings, position, and angulation between the right and left bilateral third molars in 342 patients from a Turkish population. Our study extends this earlier work by evaluating Winter angulation, the combined Pell–Gregory classification, and the Pederson difficulty index together. It also assesses right–left agreement using both raw agreement rates and kappa coefficients corrected for chance agreement.

Mesioangular angulation was the most common Winter category on both the right (45.25%) and left (38.25%) sides. When both sides were considered together, mesioangular angulation remained the most frequent at 41.75%, while IIB (32.375%) and IIA (29.0%) were the most common Pell–Gregory combinations. Santos et al. [18], whose study was not limited to impacted teeth, reported the same proportion of mesioangular angulation (41.8%) and identified IIB as the most common Pell–Gregory combination. In a study of 1000 patients with bilateral impacted mandibular third molars, Patel et al. [14] found mesioangular angulation to be predominant on both sides, with Position B and Class II being the most common depth and ramus relationship categories, respectively. Similarly, Jaroń and Trybek [1], Khojastepour et al. [5], and Akarslan and Kocabay [3] reported mesioangular angulation as the predominant category. In contrast, Yılmaz et al. [19] found vertical angulation and Position C to be the most common categories in a sample from Central Anatolia, Türkiye, while Edouma et al. [20] reported Position A as the most common depth category in a Cameroonian sample. These differences between studies may be related to variations in inclusion criteria, angulation thresholds, age distributions, and the populations examined.

When the categorical distributions on the right and left sides were compared, a significant difference was found only for Winter angulation. Mesioangular angulation was more common on the right, whereas horizontal angulation was more common on the left. Akarslan and Kocabay [3] also reported a side-to-side difference in angulation among bilateral impacted mandibular third molars. However, in contrast to our findings, horizontal angulation was more frequent on the right than on the left (40.8% vs. 25.4%), while mesioangular angulation was more frequent on the left than on the right (57.7% vs. 42.3%). Patel et al. [14], on the other hand, found no significant difference in Winter angulation between the two sides in their study of 1000 bilateral cases. These inconsistent findings suggest that there may be no reproducible side-specific pattern of angulation. The differences between studies may be related to sample characteristics, the two-dimensional nature of panoramic radiography, and variation in the assessment of cases close to classification thresholds. In the Pederson index, horizontal angulation is assigned one point more than mesioangular angulation. The higher frequency of horizontal teeth on the left may therefore have contributed to the slightly higher total Pederson score on that side. However, the mean difference between the two sides was only 0.15 points. Because intraoperative difficulty and surgical outcomes were not evaluated, the clinical relevance of this difference remains uncertain.

Similarity in the overall category distributions between the two sides and bilateral agreement within the same patient are distinct concepts. Although the overall distributions of Pell–Gregory ramus relationship, Pell–Gregory depth, and Pederson difficulty categories did not differ significantly between the right and left sides, within-patient agreement for these variables ranged from moderate to substantial. For Winter angulation, the overall distributions differed between the two sides, but within-patient agreement was still moderate. Therefore, similar category frequencies at the sample level do not necessarily mean that the right and left mandibular third molars of the same patient fall into the same category. Right–left differences in one direction in some patients may be offset by differences in the opposite direction in others, making the overall distributions appear similar. Unlike previous studies that compared only side-specific distributions [3,14], our study assessed within-patient agreement using kappa coefficients and cross-tabulations. This distinction shows the importance of evaluating agreement at the patient level. Although raw agreement for the Pederson difficulty category was 69.5%, the kappa coefficient remained moderate at 0.43 after correction for chance agreement. This may be related to the concentration of most cases in the moderate difficulty category, as kappa is known to be sensitive to imbalanced category distributions and may be relatively low despite high raw agreement [21].

It is also noteworthy that right–left disagreements were concentrated between certain categories. Nearly half of the Winter disagreements occurred between the mesioangular and vertical categories. Similarly, 41.5% of the Pell–Gregory disagreements occurred between Positions A and B within the same ramus class. In contrast, markedly different combinations, such as distoangular and horizontal angulations, were rare. An easy–difficult discrepancy was observed in only seven patients (1.75%), showing that marked differences in radiographic difficulty between the two sides were uncommon. These findings show that most differences occurred between neighboring categories. Small angular or linear differences near classification thresholds may place otherwise similar teeth in different categories. Thus, some of the observed disagreements may reflect the categorical nature of these systems rather than substantial anatomical differences. Cases close to classification thresholds may also be more affected by observer variability. Almendros-Marqués et al. [22] reported intraobserver agreement of over 85% for the Winter classification, but only 66% for the Pell–Gregory position classification. They concluded that the reproducibility of the Pell–Gregory classification was limited.

The two-dimensional nature of panoramic radiography may also contribute to uncertainty in classification. Panoramic radiography may produce different results from cone–beam computed tomography, particularly for the Pell–Gregory classification [10]. These classification systems also do not show the buccolingual position of the tooth [5]. Three-dimensional imaging may therefore change the treatment plan in some cases [23]. Some of the disagreements in our study may represent true anatomical asymmetry, while others may result from imaging and classification variability. Because of the retrospective design and the lack of recorded repeated observer assessments, we could not distinguish between these factors.

Separate analysis of the Pell–Gregory components showed different levels of agreement for ramus relationship and depth. The kappa estimate was higher for ramus relationship (κ = 0.64; 95% CI, 0.56–0.72) than for depth (κ = 0.41; 95% CI, 0.34–0.49). This difference may be because ramus relationship is based on broader skeletal features, such as the retromolar space. Depth assessment may be more affected by the eruption stage of the tooth and the identification of the occlusal plane on panoramic radiographs. Third molar position has been associated with dental arch features such as arch length and intermolar width [12]. Ramus relationship may also change with age [24]. These studies suggest that third molar position may continue to change during young adulthood. However, they do not directly explain the difference in bilateral agreement found in our study. Therefore, our explanation for the higher agreement in ramus relationship should be considered a biological interpretation. In addition, the distinction between Positions A and B depends on identifying the occlusal plane on panoramic radiographs. This may make the assessment more sensitive to observer variability [22].

The findings related to the Pederson index also require consideration. Raw agreement based on identical total scores was the lowest among the measures examined (48.75%). However, the right and left scores were either identical or differed by only 1 point in 83.25% of patients. Quadratic weighted kappa, which considers the difference between scores, was 0.52 and indicated moderate agreement. The Pederson scores were statistically significantly higher on the left side, yet the magnitude of this difference was small. The mean side difference was only 0.15 points. The corresponding rank biserial correlation was 0.19, which indicates a small effect. This means that a statistically significant side difference should not necessarily be interpreted as clinically meaningful. The total Pederson score has more possible values than the other classifications. This may have contributed to the relatively low rate of exact score agreement. In addition, among the 195 patients with the same total Pederson score on both sides, 25 (12.82%) differed in Winter angulation, the combined Pell–Gregory classification, or both. This finding shows that the Pederson score may not reflect radiographic differences between the two sides when different angulation and position combinations produce the same total score. Thus, agreement in the composite score should not be interpreted as agreement in the radiographic characteristics from which that score was derived.

The ability of the Pederson index to predict actual surgical difficulty is also debated. Diniz-Freitas et al. [25] reported that the index failed to correctly identify more than 75% of difficult extractions. García et al. [26] found that the Pell–Gregory classification, which is one component of the index, was not reliable for predicting surgical difficulty. A meta-analysis by Bali et al. [9] also concluded that the Pederson index alone was not an adequate predictive tool. In contrast, Simons et al. [27] found a significant association between the Pell–Gregory classification and the surgeon’s choice of coronectomy or surgical extraction. No such association was found for the Winter classification. Although this finding links radiographic classification to treatment selection in that study, it does not establish the clinical consequences of bilateral classification differences observed in our sample. However, the sample of Simons et al. [27] consisted of third molars closely related to the inferior alveolar nerve and referred for extraction. Their findings should therefore not be directly generalized to our sample. A recent systematic review of systematic reviews on coronectomy emphasized the importance of consistent radiographic assessment and long-term follow-up for retained-root migration, exposure, and possible re-intervention. These considerations place radiographic classifications within a broader clinical assessment rather than supporting their use alone to determine treatment [28]. Actual treatment decisions and surgical outcomes were also not evaluated in our study.

The main clinical implication of our findings is that a mandibular third molar on one side should not be used as a direct indicator for the tooth on the other side. The right and left teeth were classified differently in at least one assessment system in 230 patients (57.5%). Complete bilateral agreement across all three systems was found in 170 patients (42.5%). However, most disagreements occurred between neighboring categories. The Pederson scores were identical or differed by only 1 point in 83.25% of patients. In addition, an easy–difficult discrepancy was observed in only seven patients (1.75%). The predominance of disagreements between adjacent categories suggests that bilateral classification differences should not, by themselves, be interpreted as evidence of major differences in surgical difficulty. Therefore, each mandibular third molar should be assessed separately during radiographic evaluation and the planning of possible surgical treatment. Findings from one side should not be directly generalized to the other, particularly in cases close to classification thresholds.

This study has several strengths. It included a relatively large sample of patients with bilateral mandibular third molars. Three classification systems were evaluated together in the same patients. Right–left agreement was reported using kappa coefficients corrected for chance agreement, together with their confidence intervals. The components of the Pell–Gregory classification were also examined separately, and disagreement patterns were presented in detail using cross-tabulations. However, the retrospective, single-center design and recruitment from a university clinic limit the generalizability of the findings to populations in other clinical settings. Requiring the radiographic presence of all 32 permanent teeth may have favored the selection of younger individuals with complete dentitions. This selection criterion also limits the generalizability of the findings to patients with missing teeth. Most patients were also in the younger age groups, and the age distribution was not homogeneous. Third molar position may change over time, and the degree of bilateral agreement may differ in older patients [24]. Given the predominantly young age distribution of our sample, the applicability of these findings to older populations should be interpreted with caution. Differences in bilateral agreement according to age and sex were not examined separately.

Because the assessments were based on two-dimensional panoramic radiographs, the buccolingual position and root morphology of the teeth could not be examined. The classifications were established through joint assessment and consensus, with unresolved cases adjudicated by a senior faculty member. Because separate independent ratings were not recorded, interobserver reliability could not be quantified. However, interobserver reliability was not calculated separately. The observed bilateral disagreement may reflect a combination of true anatomical asymmetry, limitations of two-dimensional panoramic imaging, and observer-related classification variability. The relative contributions of these factors could not be determined in this study. The radiographic classifications were also not compared with intraoperative difficulty, surgical duration, or complications. Our findings are therefore limited to radiographic agreement. Because clinical eruption status was not recorded separately, its potential influence on classification distributions, Pederson scores, and bilateral agreement could not be assessed. A further limitation is that no a priori sample size calculation was performed. The sample size was determined by the available consecutive patients rather than a prespecified statistical power calculation. Future studies should measure interobserver reliability, use cone–beam computed tomography images obtained for clinical indications, and examine changes in bilateral agreement with age. Radiographic agreement should also be compared with actual surgical outcomes.

## 5. Conclusions

Side-specific category distributions did not fully reflect patient-level bilateral agreement in mandibular third-molar classifications. Bilateral mandibular third molars showed moderate right–left agreement in Winter angulation, the Pell–Gregory classification, and the Pederson difficulty index. Complete bilateral agreement across all three assessment systems was found in only 42.5% of patients. Among the Pell–Gregory components, ramus relationship showed substantial agreement, while most disagreements involved the depth component. Disagreements in Winter angulation occurred most often between the mesioangular and vertical categories. The Pederson score was significantly higher on the left, although the mean difference between the two sides was only 0.15 points. These findings suggest that radiographic characteristics on one side should not be directly generalized to the other. Accordingly, each mandibular third molar should be assessed independently during radiographic evaluation and surgical planning.

## Figures and Tables

**Figure 1:**
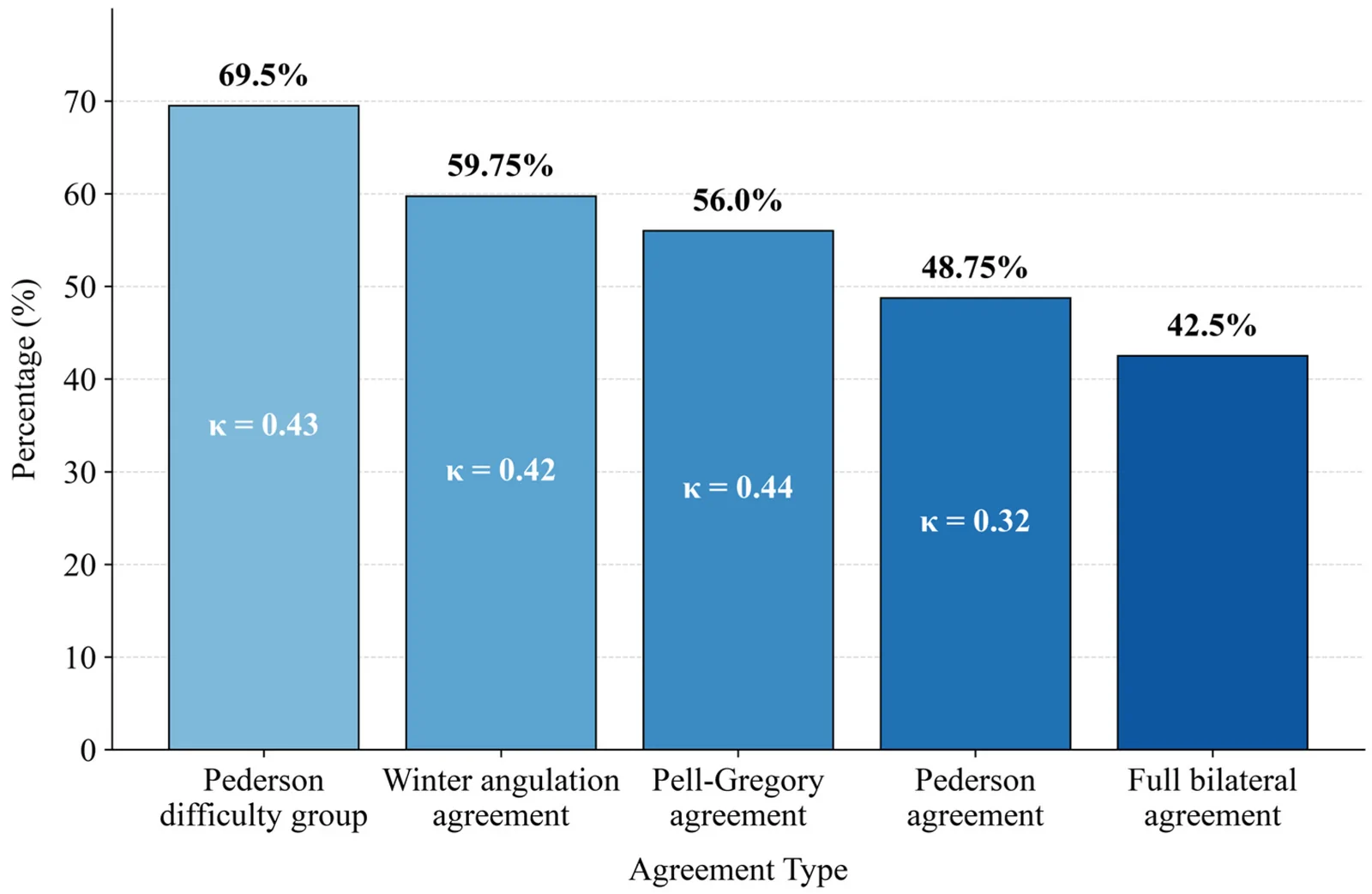
Raw bilateral agreement rates and Cohen’s kappa coefficients between the right and left mandibular third molars for each assessment system. The rightmost bar shows the proportion of patients with complete bilateral agreement across Winter angulation, the Pell–Gregory classification, and the Pederson score (*n* = 400).

**Figure 2:**
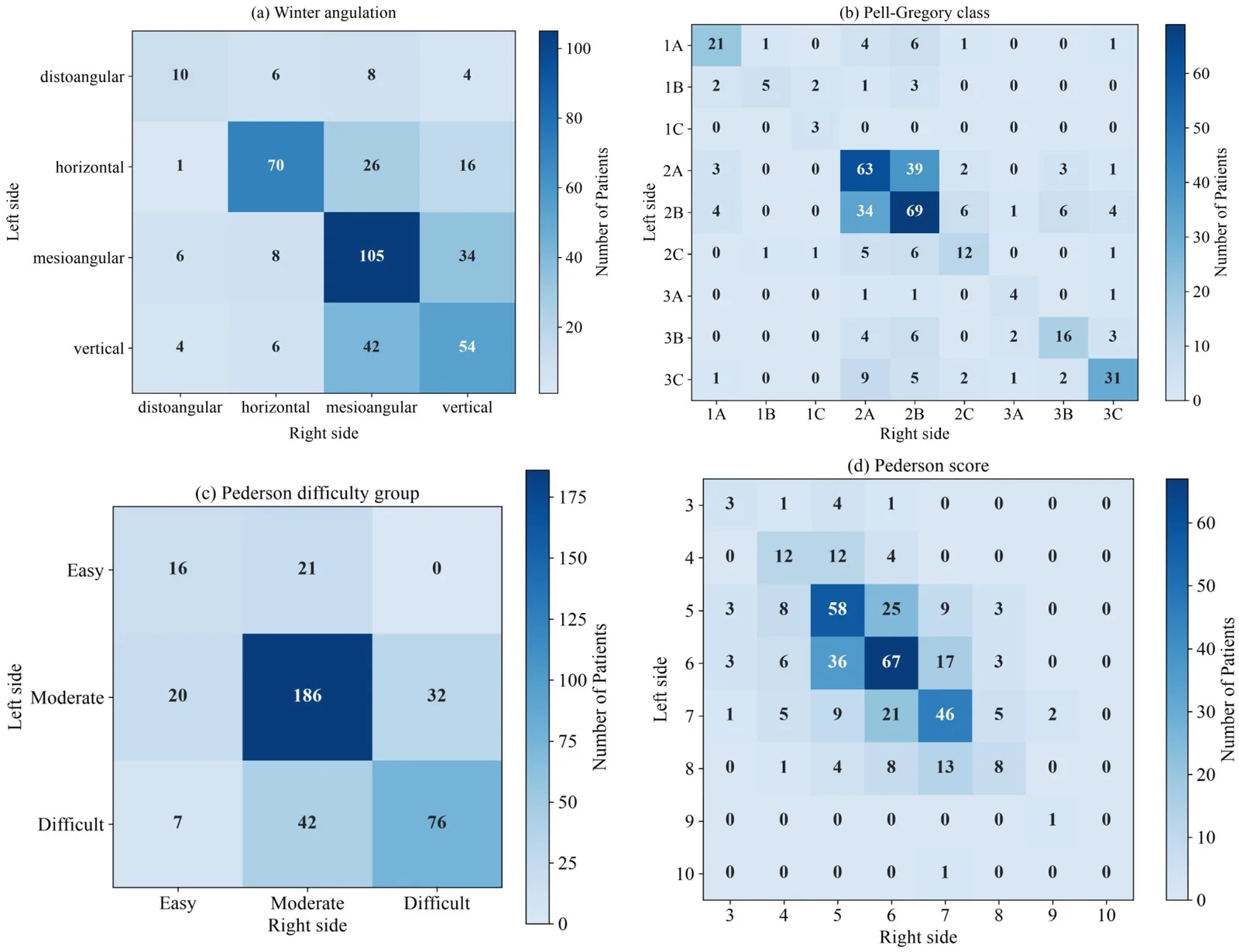
Cross-tabulations of the left (rows) and right (columns) mandibular third molars: (**a**) Winter angulation, (**b**) combined Pell–Gregory classification, (**c**) Pederson difficulty category, and (**d**) Pederson score. Bold diagonal cells indicate patients with bilateral agreement; cell values represent the number of patients.

**Table 1:** Radiographic classification criteria for mandibular third molars and the Pederson difficulty index.

Classification	Category	Radiographic Definition	Pederson Score
Winter Angulation	Mesioangular	The long axis of the third molar is inclined mesially relative to that of the second molar	1
	Horizontal	The long axis of the third molar is oriented horizontally relative to that of the second molar	2
	Vertical	The long axis of the third molar is approximately parallel to that of the second molar	3
	Distoangular	The long axis of the third molar is inclined distally relative to that of the second molar	4
Pell–Gregory Classification: Depth	Position A	The occlusal plane of the third molar is at the same level as that of the second molar	1
	Position B	The occlusal plane of the third molar lies between the occlusal plane and the cervical line of the second molar	2
	Position C	The third molar is positioned below the cervical line of the second molar	3
Pell–Gregory Classification: Ramus Relationship	Class I	The space between the distal surface of the second molar and the anterior border of the ramus is sufficient to accommodate the mesiodistal width of the third molar	1
	Class II	The space between the distal surface of the second molar and the anterior border of the ramus is less than the mesiodistal width of the third molar	2
	Class III	All or most of the third molar is located within the mandibular ramus	3
Pederson Difficulty Index	Easy	Total Pederson score: 3–4	
	Moderate	Total Pederson score: 5–6	
	Difficult	Total Pederson score: 7–10	

Pederson difficulty score was calculated by summing the scores assigned for Winter angulation, Pell–Gregory depth, and ramus relationship. Total scores of 3–4, 5–6, and 7–10 were classified as easy, moderate, and difficult, respectively.

**Table 2:** Demographic characteristics of the study population.

Variable	Value
Sex	*n* (%)
Female	268 (67.0%)
Male	132 (33.0%)
Age	Years
Mean ± SD	22.2 ± 4.4
Median (IQR)	22 (19–24)
Range (min–max)	11–48
Age Group	*n* (%)
≤20	150 (37.5%)
21–30	230 (57.5%)
31–40	19 (4.75%)
≥41	1 (0.25%)

SD: Standard deviation; IQR: interquartile range.

**Table 3:** Distribution of Winter angulation, Pell–Gregory classification, and Pederson difficulty index for the right and left mandibular third molars.

Classification	Category	Right, *n* (%)	Left, *n* (%)	Total, *n* (%)
Winter Angulation	Mesioangular	181 (45.25%)	153 (38.25%)	334 (41.75%)
	Vertical	108 (27.0%)	106 (26.5%)	214 (26.75%)
	Horizontal	90 (22.5%)	113 (28.25%)	203 (25.375%)
	Distoangular	21 (5.25%)	28 (7.0%)	49 (6.125%)
Pell–Gregory: Ramus	Class I	44 (11.0%)	50 (12.5%)	94 (11.75%)
	Class II	279 (69.75%)	261 (65.25%)	540 (67.5%)
	Class III	77 (19.25%)	89 (22.25%)	166 (20.75%)
Pell–Gregory: Depth	Position A	160 (40.0%)	152 (38.0%)	312 (39.0%)
	Position B	169 (42.25%)	168 (42.0%)	337 (42.125%)
	Position C	71 (17.75%)	80 (20.0%)	151 (18.875%)
Pell–Gregory: Combined	IA	31 (7.75%)	34 (8.5%)	65 (8.125%)
	IB	7 (1.75%)	13 (3.25%)	20 (2.5%)
	IC	6 (1.5%)	3 (0.75%)	9 (1.125%)
	IIA	121 (30.25%)	111 (27.75%)	232 (29.0%)
	IIB	135 (33.75%)	124 (31.0%)	259 (32.375%)
	IIC	23 (5.75%)	26 (6.5%)	49 (6.125%)
	IIIA	8 (2.0%)	7 (1.75%)	15 (1.875%)
	IIIB	27 (6.75%)	31 (7.75%)	58 (7.25%)
	IIIC	42 (10.5%)	51 (12.75%)	93 (11.625%)
Pederson Difficulty	Easy (3–4)	43 (10.75%)	37 (9.25%)	80 (10.0%)
	Moderate (5–6)	249 (62.25%)	238 (59.5%)	487 (60.875%)
	Difficult (7–10)	108 (27.0%)	125 (31.25%)	233 (29.125%)

Percentages were calculated separately for each side based on 400 teeth. The right- and left-side distributions were compared using the Stuart–Maxwell test of marginal homogeneity: Winter angulation, *p* = 0.006; Pell–Gregory ramus relationship, *p* = 0.092; Pell–Gregory depth, *p* = 0.419; and Pederson difficulty category, *p* = 0.135.

**Table 4:** Bilateral agreement between the right and left mandibular third molars: raw agreement rates and Cohen’s kappa coefficients.

Assessment System	Patients with Agreement *n* (%)	Unweighted κ (95% CI)	Linearly Weighted κ (95% CI)	Quadratically Weighted κ (95% CI)	Level of Agreement *
Winter angulation	239 (59.75%)	0.42 (0.35–0.48)	—	—	Moderate
Pell–Gregory combined	224 (56.0%)	0.44 (0.37–0.50)	—	—	Moderate
Ramus relationship (I–III)	330 (82.5%)	0.64 (0.56–0.72)	—	—	Substantial
Depth (A–C)	251 (62.75%)	0.41 (0.34–0.49)	—	—	Moderate
Pederson score (3–10)	195 (48.75%)	0.32 (0.26–0.39)	0.42 (0.36–0.49)	0.52 (0.44–0.59)	Fair (unweighted); moderate (weighted)
Pederson Difficulty Group	278 (69.5%)	0.43 (0.35–0.51)	0.46 (0.38–0.54)	—	Moderate
Complete Bilateral Agreement **	170 (42.5%)	—	—	—	—

κ: Cohen’s kappa. CI: confidence interval. * According to the Landis and Koch criteria (0.21–0.40, fair; 0.41–0.60, moderate; 0.61–0.80, substantial). Since the Pell–Gregory and Winter classifications are nominal, only unweighted kappa coefficients were calculated. ** Complete bilateral agreement across all systems (Winter + Pell–Gregory + Pederson score).

## Data Availability

The data are not publicly available due to patient privacy and ethical restrictions. De-identified data may be made available by the corresponding author upon reasonable request and subject to institutional approval.
